# Complete mitochondrial genome of bronze-winged parrot (*Pionus chalcopterus chalcopterus*, Psittaciformes)

**DOI:** 10.1080/23802359.2017.1390404

**Published:** 2017-10-17

**Authors:** Adam Dawid Urantówka, Aleksandra Kroczak, Paweł Mackiewicz

**Affiliations:** a Department of Genetics, Wroclaw University of Environmental and Life Sciences, Wroclaw, Poland;; b Department of Genomics, Faculty of Biotechnology, Wrocław University, Wrocław, Poland

**Keywords:** Androglossini, bronze-winged parrot, mitogenome, *pionus chalcopterus chalcopterus*, psittaciformes

## Abstract

Medium-sized neotropical parrots from *Pionus* genus are represented by at least eight species. However, their taxonomy should be revised because some external morphological characters together with genetic data recognize 19 taxa. At present, only two mitochondrial markers are available for most of these taxa and obtained phylogenies are not well resolved. Therefore, we sequenced *Pionus chalcopterus chalcopterus* mitogenome to gain more molecular data required for future studies of the taxonomical status and phylogenetic relationships between *Pionus* taxa. Performed phylogenetic analyses showed seven monophyletic clades including at least two sequences assigned to one species. However, not all subspecies sequences were monophyletic.

*Pionus* parrots are diversified into many species and subspecies inhabiting various environments, from mountains to lowland with dry- and wet-forests (Forshaw 2010). Therefore, they offer an interesting possibility to study mechanisms of speciation and emergence of subspecies. Phylogenetic analyses showed independent biogeographic disjunctions of the lineages occupying the same types of habitats. The diversification of these parrots was associated with the Andes uplifted and Pleistocene climatic oscillations (Ribas et al. 2007). The study was based only on two mitochondrial gene sequences. Therefore, to enrich the set of molecular markers, we obtained the sequence of mitochondrial genome from *Pionus chalcopterus* (accession number MF784450). In comparison to the firstly sequenced mitogenome from *Pionus menstruus* (Urantówka and Mackiewicz 2016), *P. chalcopterus* mitogenome differs in the length of tRNA-Phe and 16S rRNA genes as well as two control regions. Moreover, the stop codon in *nd5* gene lacks the last nucleotide in *P. chalcopterus*, which may be associated with the loss of the 10-bp intergenic region between *nd5* and *cytb*.

Morphology of the analyzed parrot is typical of *chalcopterus* species and subspecies. The taxonomic position of this species is undoubtedly proved in the phylogenetic tree of *nd2* + *cytb* alignment including all available *Pionus* taxa (Figure 1). The specimen groups significantly within three other representatives of its species. Seven clades including at least two sequences assigned to one species can be recognized as monophyletic in the tree. Five of them (*menstruus*, *chalcopterus*, *seniloides*, *fuscus*, *maximiliani*) form very significantly supported clades by Bayesian and maximum likelihood (ML) methods. The clade of *senilis* obtained the highest posterior probability but moderate bootstrap support, whereas *sordidus* clade is poorly supported by the both methods. All three subspecies of *P. menstruus* are represented by at least four samples create monophyletic groups. However, two *P. chalcopterus chalcopterus* sequences are not clustered together and one of them groups significantly with two *P. chalcopterus cyanescens* sequences. The group of three *P. maximiliani lacerus* sequences is not fully monophyletic either, because this clade includes also *P. maximiliani siy*. This may result from low variation of the studied markers, misidentification of subspecies or mitochondrial DNA introgression. A study of more variable control region can solve this controversy.

**Figure 1.**
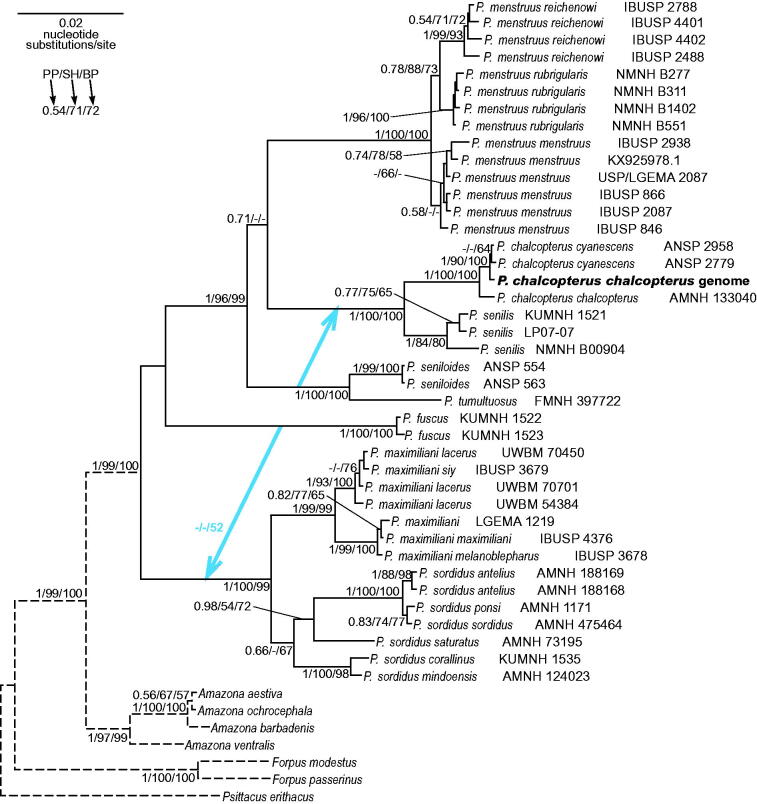
The phylogenetic tree obtained in MrBayes for the concatenated alignment of *nd2* and *cytb* genes (2181 bp) indicating that the studied individual (**bolded**) belongs to *Pionus chalcopterus* species. The individual is a bird kept in culture in Poland (Gorzyce town). Its blood sample, from which DNA was isolated, is available in the collection of the laboratory at the Department of Genetics in Wroclaw University of Environmental and Life Sciences under the number ADUAKPM07. The blue arrows indicate alternative positions of clades in the topology received in IQ-TREE. The length of branches leading to outgroup sequences was shortened five times (the dashed lines). Values at nodes, in the order shown, indicate posterior probabilities found in MrBayes (PP) as well as SH-aLRT (SH) and non-parametric bootstrap (BP) support percentages calculated in IQ-TREE. The posterior probabilities <0.5 and the percentages <50% were omitted or indicated by a dash ‘–’. In the MrBayes (Ronquist et al. 2012) analysis, we assumed separate mixed substitution models for three codon positions in these genes of six possible partitions, with information about heterogeneity rate across sites as proposed by PartitionFinder (Lanfear et al. 2012). We applied two independent runs, each using four Markov chains. Trees were sampled every 100 generations for 20,000,000 generations. After obtaining the convergence, trees from the last 9,452,000 generations were collected to compute the posterior consensus. In the case of IQ-TREE (Nguyen et al. 2015), we used separate nucleotide substitutions models for four partitions as suggested by ModelFinder (Chernomor et al. 2016; Kalyaanamoorthy et al. 2017). In SH-aLRT bootstrap analysis, 10,000 replicates were assumed, and in non-parametric bootstrap, 1000 replicates were applied. All sequences passed the composition χ^2^ test. The analyzed sequences were downloaded from GenBank database (/www.ncbi.nlm.nih.gov) under the accession numbers: AY669403, AY669447, DQ143290, DQ143304, EF517606-24, EF517628-71, HM755882, HQ270500, JX524615, KM611467, KM611470, KM611474, KT361659, KX925977/78.

The deep branches of the tree are generally well supported with the exception of two relationships marked by blue arrows in Figure 1. Two applied methods provided alternative topologies but none of them was significantly favoured. Interestingly, the clustering the clade of *P. seniloides* +* P. tumultuosus* with the clade of *P. chalcopterus* +* P. senilis*, as proposed by the ML tree, agrees with the maximum parsimony tree when morphological characters were added to the molecular data (Ribas et al. 2007). In fact, all these taxa differ from other *Pionus* in entirely yellow bill and they also have white throat with the exception to *P. tumultuosus*. The analyses of complete mitochondrial genomes are necessary to fully resolve the relationships because results for individual markers can be biased and produce inconsistent phylogenies of parrots (Urantówka et al. 2017).

## References

[CIT0001] Chernomor O, von Haeseler A, Minh BQ. 2016 Terrace aware data structure for phylogenomic inference from supermatrices. Syst Biol. 65:997–1008. 27121966 10.1093/sysbio/syw037 PMC5066062

[CIT0002] Forshaw JM. 2010 Parrots of the world. London: A & C Black Publishers Ltd; p. 328.

[CIT0003] Kalyaanamoorthy S, Minh BQ, Wong TKF, von Haeseler A, Jermiin LS. 2017 ModelFinder: fast model selection for accurate phylogenetic estimates. Nature Methods. 14:587–589. 28481363 10.1038/nmeth.4285 PMC5453245

[CIT0004] Lanfear R, Calcott B, Ho SY, Guindon S. 2012 Partitionfinder: combined selection of partitioning schemes and substitution models for phylogenetic analyses. Mol Biol Evol. 29:1695–1701. 22319168 10.1093/molbev/mss020

[CIT0005] Nguyen LT, Schmidt HA, von Haeseler A, Minh BQ. 2015 IQ-TREE: a fast and effective stochastic algorithm for estimating maximum-likelihood phylogenies. Mol Biol Evol. 32:268–274. 25371430 10.1093/molbev/msu300 PMC4271533

[CIT0006] Ribas CC, Moyle RG, Miyaki CY, Cracraft J. 2007 The assembly of montane biotas: linking Andean tectonics and climatic oscillations to independent regimes of diversification in Pionus parrots. Proc Biol Sci. 274:2399–2408. 17686731 10.1098/rspb.2007.0613 PMC2274971

[CIT0007] Ronquist F, Teslenko M, Van Der Mark P, Ayres DL, Darling A, Höhna S, Larget B, Liu L, Suchard MA, Huelsenbeck JP. 2012 MrBayes 3.2: efficient bayesian phylogenetic inference and model choice across a large model space. Syst Biol. 61:539–542. 22357727 10.1093/sysbio/sys029 PMC3329765

[CIT0008] Urantówka AD, Kroczak A, Mackiewicz P. 2017 The influence of molecular markers and methods on inferring the phylogenetic relationships between the representatives of the Arini (parrots, Psittaciformes), determined on the basis of their complete mitochondrial genomes. BMC Evol Biol. 17:166. 28705202 10.1186/s12862-017-1012-1 PMC5513162

[CIT0009] Urantówka AD, Mackiewicz P. 2016 The first complete mitochondrial genome sequence from the blue-headed parrot (*Pionus menstruus menstruus*): a representative for the genus. Mitochondrial DNA Part B. 1:891–892. 10.1080/23802359.2016.1258341 PMC7800462 33473668

